# The new "Tehran Back Belt": Design then testing during a simulated sitting task improved biomechanical spine muscle activity

**DOI:** 10.15171/hpp.2019.16

**Published:** 2019-05-25

**Authors:** Hamidreza Mokhtarinia, Javad Ghamary, Azam Maleki-Ghahfarokhi, Morteza Asgari, Charles Philip Gabel, Mohamad Parnianpour

**Affiliations:** ^1^Department of Ergonomics, University of Social Welfare and Rehabilitation Sciences, Tehran, Iran; ^2^Student Research Committee, Faculty of Health, Tabriz University of Medical Sciences, Tabriz, Iran; ^3^Department of Mechanical, Aerospace and Biomedical Engineering, University of Tennessee, Knoxville, USA; ^4^Independent Researcher, Coolum Beach, Qld, Australia; ^5^Department of Mechanical Engineering, Sharif University of Technology, Tehran, Iran

**Keywords:** Back belt, Para-spinal muscle activity, Sitting posture, User experience

## Abstract

**Background:** Spinal load and muscle activity in occupation settings is an area of increasing concern. Regarding technological advancements, in diverse occupations the spinal loads have increased through constrained seated postures. Back belts are consequently used in prophylactic and conservative management of occupational low back pain (LBP) in two distinct settings, prevention in industry, and treatment in LBP management. Industrial sites utilize belts for LBPprophylaxis on a large scale with their design and user experience (UE) influencing both the effectiveness and the workers’ compliance. This pilot study aims at determining the effectiveness of the new Tehran Back Belt (TBB) and assesses both UE and biomechanical effect (BE) on para-spinal muscle activity in healthy subjects.

**Methods:** A pretest-posttest study. Stage-1, design and fabrication of the TBB. Stage-2, the UE of the designed belt evaluated in healthy volunteers (n=30) via a checklist. The BE was determined from the level of lumbar extensor and trunk flexor muscle activity gauged during two test conditions of sitting posture (with and without belt) over 35-minute periods.

**Results: ** Most subjects (>90%) reported high ‘ease of use’ and ‘comfort’ while wearing the TBB.The BE statistical analysis showed significantly reduced EMG activity levels for the longissimus(P = 0.012, η^2^=0.24), rectus abdominis (P=0.024, η^2^=0.18) and internal oblique (P=0.001,η^2^=0.44) muscles in belt-use conditions.

**Conclusion: ** Decreased muscle activity while using the TBB is potentially advantageous for workers as spinal muscle activity is significantly reduced. Further investigations for longer duration effects and during real work office-based activities are warranted.

## Introduction

Low back pain (LBP) is the most prevalent occupational disease in people under 45 years of age.^1-3^ The economic burden associated with LBP embraces both the direct costs, such as medical care and compensation for lost wages, and indirect costs, such as recruiting and training new staff and related productivity losses.^2,4-6^ The incidence of LBP leads to reduced efficiency and productivity in organizations and enormous costs to the health systems.^7,8^

Numerous risk factors contribute to the causes of LBP including provocative and prolonged sitting postures.^9-11^ In recent decades, particularly owing to technology advancements, numerous jobs and occupations are increasingly performed in constrained seated postures.^12^ Various sitting postures attempt to accommodate the demands and include: flat, slump, long and short lordosis.^13^ Biomechanical studies have noted that muscular activity is lowest in the flat position and respectively augmented in the remaining three positions.^14^ Furthermore, intra-discal pressure (IDP) varies with posture and position. It is reported as lowest in supine positions, intensifying in standing and at a maximum level in sitting. Among sitting postures, ‘flat’ has resulted in the lowest IDP.^15-17^ Adopting and maintaining this flat posture while seated, particularly for prolonged periods, is often difficult. Although a variety of workplace interventions have attempted to attenuate this, including ergonomic desks and chairs,^18^ it has not declined.^19^ One of the assistive devices that gained popularity in recent years is back belt supports. Currently, many industrial work settings have introduced back belts for LBP prophylaxis on a large scale,^20,21^ but many employees do not use them due to discomfort. Besides their use as a preventive measure, lumbar supports are also used in the treatment of patients with LBP. The suggested mechanism effects are increased intra-abdominal pressure, enhanced torso muscle support and decreased spine and torso mobility.^22-24^ Both reduced muscle fatigue and reduced compressive loading may result in a decrease of the risk for LBP.^22,23^ Another indication of back belt effectiveness can be maintaining the lumbar lordosis during the sitting posture and related reductions in spine muscle activity which appear neglected in most recent research.^23,25^ Chen^25^ introduced a back belt in order to maintain the lumbar lordosis but it had limitations such as low user comfort, limitations of knee range of motion, applying pressure on the knee and reduced movement freedom.

It appears that design and usability have an important role in the effectiveness, acceptance and adoption of back braces in the workplace, including offices and industrial settings. The present study was conducted with the aim of designing and biomechanically evaluating a new ergonomic back belt, the Tehran Back Belt (TBB). The TBB user experience (UE), defined as “a person’s perceptions and responses that result from using or anticipated use of a product, system or service”^26^ was evaluated. The biomechanical effect (BE) was also evaluated on the para-spinal muscle activity in healthy subjects. Feedback on effectiveness, user acceptance and potential adoption in the workplace was assessed and recommendations for further investigation determined.

## Materials and Methods

### Participants

Healthy male students were recruited from a sample of convenience at the University of Social Welfare and Rehabilitation Sciences (USWR) in Tehran during a six-month period from December 2016 to May 2017. Participants’ (n = 30, age = 23.2±1.4 years, height = 174.7±4.5 cm, weight = 71.1±4.7 kg and body mass index (BMI) = 23.3±1.5 kg/m^2^) with inclusion criteria being willingness to consent to participate and exclusion criteria being a history of LBP and neuro-muscular or orthopaedic dysfunction. An experienced physiotherapist undertook a physical examination to ensure participants had no abnormal restriction in hip or spinal mobility or the presence of scoliosis in order to minimize potential constraints on the symmetrical performance of sitting postures.

All subjects read and signed an informed consent and ethics was provided by the ethics committee of the USWR.

### Methods

*Back belt design and feedback questionnaire*

**Figure 1:**
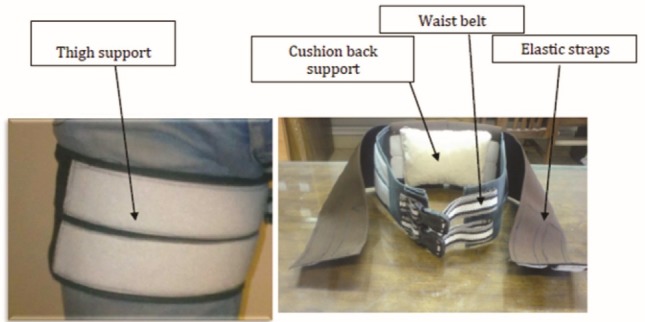
The new back belt (TBB) and its components proposed by this study.

**Figure 2:**
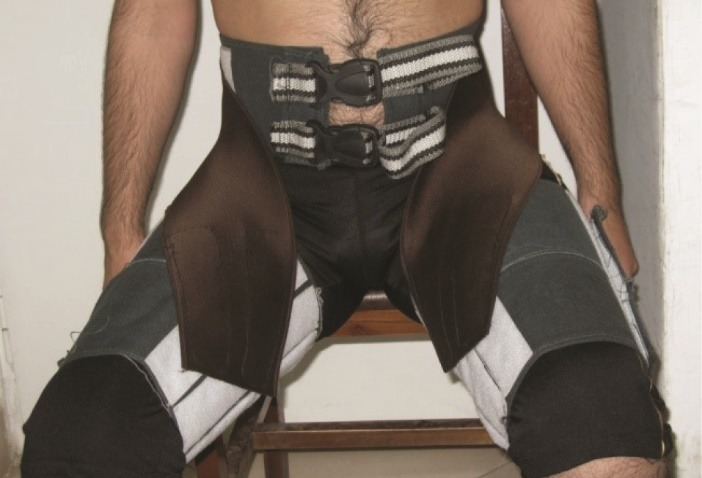
The elastic straps.

This study was conducted in two stages, design of the TBB, and testing and analysis for UE and BE. Stage-1 designed the TBB with four main parts: a waist belt, a cushioned back support, two thigh-support portions and two elastic straps. The TBB was designed and fabricated specifically for this study as an evolutionary progression on existing designs that utilized both extensible and non-extensible features (Figure 1).^27^ The initial two components are worn respectively around the waist and on the thighs. These portions are composed of stiff plastic and secured with Velcro fasteners respectively at the waist and thighs. The third component, elastic straps, connects the waist belt to the thigh portions bilaterally. These straps attach to the waist portion in the lumbar region then extend diagonally in front of the thighs and attach to the inner sides of the thigh-support portions of the belt (Figure 2). The fourth component, the cushion back support, is located inside the waist portion and supports the lumbar lordosis.

Additionally, a checklist with the aim of UE of the TBB evaluation was developed. The UE is the ‘…practice of enhancing user satisfaction with a product by refining the convenience and pleasure provided in contact with the product’. According to this assumption, the constructed checklist should contain two classes of items: a) items which measure the perceived comfort and ease of use directly, and b) items which measure the quality of the product on the relevant aspects.^28^

Two brainstorming sessions (each lasting 90 minutes) with nine professionals: industrial design (n = 2), biomechanics (n = 2), physiotherapy (n = 1), and ergonomics (n = 4) were conducted. The experts were asked to propose terms they considered to be characteristic for the assessment of UE. The initial list included a total of 35 items and characteristics. The common items were reduced and deleted and the consolidated list consisted of 16 items. Five usability experts then individually extracted 13 of the 16 terms. These 13 items were again considered by the same focus group, were discussed and after considering five drafts, they agreed on a consensus version comprised of seven questions (Table 1). In a pilot study, we assessed the UE test-retest reliability in 20 students (intra-class correlation coefficient [ICC_(2,1)_] = 0.78) which indicated good reliability.

*Experimental design*

Stage-2 measured the level of para-spinal muscle activity while wearing the TBB through the use of surface electromyography (EMG) with signals collected via a Biometrics Ltd, DataLink and EMG sensors (SX230, Biometrics Ltd., Gwent, UK). The electrodes diameters were 1 cm and the center-to-center fixed inter-electrode distance was 2 cm. Before electrode placement the skin was shaved and cleaned with an alcohol swab. Three EMG sensors were placed unilaterally and parallel over the following muscles: rectus abdominis (3 cm superior to the umbilicus and 3 cm lateral to midline), external oblique (lower edge of eighth rib), internal oblique (2 cm medial and inferior to the anterior superior iliac spine), thoracic longissimus (2 cm lateral to T12 spinous process),^29^ superficial lumbar iliocostalis (3 cm lateral to midline at L2 level) and superficial lumbar multifidus (2 cm lateral to midline at the L4–L5 inter spinous space.^30,31^ The reference electrode was cited on the ulnar process.

After skin surface electrode placement and a single practice trial at maximal voluntary contraction (MVC), testing was initiated. Testing involved recording a single repetition of five seconds duration at MVC against manual resistance with activation and recordings achieved using standard protocols as follows.^32^ In the supine position, subjects were asked to flex their trunk to recruit rectus abdominis muscle, and to rotate the trunk to the left for the external oblique muscle. In the sitting position, subjects carried out a maximal forced expiratory maneuver for the internal oblique. In the prone position, subjects were requested to extend the trunk to recruit the spinal extensor muscles. In order to determine baseline amplitude activity, the electrical activity of each muscle at rest in the supine and prone positions was also recorded.

*Procedure*

Two random test trials in the sitting posture were arranged with: the belt position in which the participants were to use and wear it, and again without the belt. The experiment trials were separated by a 15-minute rest interval. In each condition, subjects were required to adopt a flat sitting posture as determined from visual inspection by the physiotherapist. A workstation including a desk, 70 cm in height accompanied by a height-adjustable stool, was provided to simulate the assembling task. Based on the standardized instructions,^13^ the participants were positioned in the flat sitting posture by the same investigator (G.J) for all trials, and performed a 35-minute puzzle montage assembly task.

Raw EMG was captured from the first 60 seconds of every five minutes, for seven periods. These were recorded at a sampling rate of 1000 Hz with a band-pass filter for the 20-450 Hz frequency range and amplification (Common Mode Rejection Ratio = 110 dB, differential amplification gain = 1000, noise <5 μV), and stored electronically for later analysis. The raw EMG data was subsequently exported to Matlab software; a 50 seconds sample was selected from each trial. Root mean square (RMS) amplitude was calculated for each trial via Matlab software. Normalization was achieved for per time period through the “equation 1”.^33^

Eq. (1) $$EMG_{norm\;}\;=\frac{EMG_{Value}-EMG_{Rest}}{EMG_{MVC}-EMG_{Rest}}$$

At the end of the trial, subjects were asked to complete the checklist relating to their subjective experience on the TBB use.

*Statistical analysis*

Descriptive statistics were used to analyze the UE checklist. A two-way repeated measure analysis of variance (ANOVA) was applied to evaluate the EMG activity of the upper body muscles at seven-time intervals (1, 5, 10, 15, 20, 25 and 30th minutes of task) between two conditions of belt and no-belt. Pairwise Sidak comparisons were performed for post-hoc analysis. Since the variables were normally distributed, the parametric tests were used.

Before conducting the ANOVA design, the normality and sphericity assumptions were monitored with K-S and Mauchly tests, respectively. Mauchly’s test of sphericity was significant and Huynh-Feldt correction was reported instead. Effect size was reported by value of the squared Eta (*η*^2^).

All statistical analyses were conducted using the IBM SPSS Statistics version 19.0 (IBM SPSS Statistics, Armonk, USA) for Windows with significance at α = 0.05.

## Results

The results UE checklist showed that >90% of subjects found the TBB had ‘ease of use’ as well as ‘being comfortable/comfortableness’. They also reported the TBB helped them maintain the flat back posture effectively. Table 1 shows descriptive statistics of the UE checklist.

**Figure 3:**
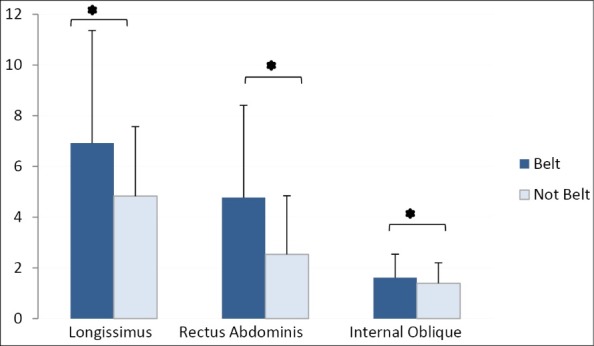
The main effect of belt conditions on longissimus, rectus abdominis, and internal oblique muscle activity.

**Figure 4:**
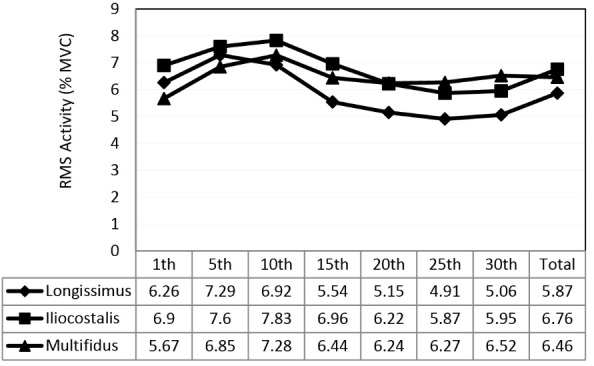
Main effect of time on muscle activities (longissimus, iliocostalis, multifidus).

The results showed normal distribution of variables (*P *> 0.05). Muscles activity level was compared in two conditions, belt and no belt, and in seven time intervals. The descriptive results of muscle activity are presented in Table 2 and results of ANOVA are provided in Table 3. Results showed that activity of longissimus, rectus abdominis, and internal oblique muscles was significantly lower during belt use (*P *< 0.05) (Figure 3). Similarly, for the External oblique muscle, lower activity was present during belt use but this was not statistically significant (*P *= 0.087). Longissimus, iliocostalis and multifidus muscle activity varied significantly with time (*P *< 0.05). Pairwise comparisons showed that for all three muscles EMG activity decreased as time progressed (Figure 4). No significant interaction of the belt by time was seen for muscle activity.

## Discussion

This prospective study produced and analyzed the TBB design and assessed the extent of the muscle activity during a simulated assembling task. Further, the UE of the TBB was evaluated. The UE can be tested via several techniques through which users interact systematically with a product under controlled conditions in order to perform a goal oriented task in an applied scenario.^34^ To date, a number of testing methods have been proposed and categorized as either subjective (e.g. using questionnaires or checklists) or objective methods (e.g. applying biomechanical techniques).^35^

We evaluated the UE of the TBB during the assembling task and consequently used the subjective testing method of a meticulously constructed checklist. As explained in the results section, a considerable number of participants (66.6%) determined the belt was appropriate for the sitting task in question. In addition, most subjects (>80%) reported the TBB as adjustable, easy to use, and that it did not limit motion during the sitting task, as well as being comfortable which increased user compliance. Effectively, they appraised the TBB as ergonomically appropriate for flat back posture maintenance during a prolonged sitting task.

Previously, Vink et al^36^ suggested a comparable back belt called ‘Back-Up’. The Back-Up had two straps around each knee connected to the back pad; therefore the straps caused pressure and discomfort in the lower extremity and the subject’s mobility was affected during use. Another proposed back belt by Chen,^25^ utilized two elastic straps along the user’s thighs and two adjustable pads which could be moved from the knee to the shanks. The advantage of the new TBB design over both these previous belts is that of greater knee freedom during use. This in turn leads to improved comfort, UE and most likely a subsequent positive effect on compliance in the real-world work setting.

Reduced longissimus, rectus abdominis, and internal oblique muscle activity during TBB use compared with a no-belt condition can be justified by the belt’s elasticity that created a cushioning effect which in-turn prevented muscle over activity during sitting. Accordingly, the belt assisted the trunk muscles and facilitated reduced activity.^25^ Another supportive mechanism is that of a sensed awareness in relation to the central nerve system. It can be hypothesized that the central nerve system awareness of the belt support component and the elastic stretch effect of the thigh portion on the lumbar region reduced muscle activity while maintaining optimal trunk posture.^37^ As detailed in the TBB design section, the back support portion was linked to the thigh portions via elastics that transferred the force to the lower legs.

This study also indicated there was no difference in the multifidus activity either with or without the TBB. This is clarified by the anatomical role of the multifidus muscle being unique as a spinal extensor muscles in its capacity to adjust and support the lumbar lordosis.^14^ Several researches have indicated that LBP subjects,^38^ post spinal trauma,^15^ and subjects with post-surgical retraction,^39^ demonstrated higher multifidus muscle activity. Claus et al^14^ argued that multifidus muscle activity level in the flat posture approached a 4% MVC. Our results demonstrated a relative similar activity level with belt usage where multifidus activity approached 6.5% MVC (Table 2). The finding of no difference in the multifidus muscle activity in the two situations of belt/no-belt may also be a consequence of higher overall multifidus activity when compared with other muscles. However, this noted activity reduction cannot be attributed solely to the belt usage. It was however reported that the TBB caused discomfort while walking. This could be potentially solved by opening the elastic straps around the thighs when subjects wanted to change from sitting to standing.

### Strengths and weaknesses of the study

Strengths of this study were that a new design and concept in bracing to reduce potential LBP was achieved successfully. The brace was trialed and found to be suited to the simulated tasks for which it is intended with reductions in lumbar specific muscle activity and a higher patient preference for its use over no support at all. A weakness of this research is that it was a simulated task being conducted in a laboratory setting on students, rather than the ideal circumstances of real workers in true occupational assembling tasks at industrial sites. Additionally, this study only used male students and the results cannot be generalized to females or whole population groups with mixed gender. Further, as a pilot study, no LBP subjects with known pathology or recognized conditions were investigated and no comparative or criterion comparison was used. However, as an investigation of the conceptual basis of the TBB as a potential intervention, ‘proof of concept’ was determined from the significant reduction in activity of the appropriate muscle and the higher patient-preference for use.

## Conclusion

This study, investigated regional muscle activity at low percentages of MVC in a task oriented sitting posture. The study sought to explore the TBB, a novel back belt designed with the aim of reducing back stress and diminishing back muscle activity during a sitting task. In addition, the user experience was quantified and assessed. The findings corroborated that wearing the belt significantly reduced para-spinal muscles activity, apart from multifidus, iliocostalis and external oblique. It appeared the TBB may provide ergonomic benefit and be considered as a beneficial assistive device to ease spinal loading in task oriented sitting postures. Consequently, it could have implications for poorly designed workstations without back support. Further research is required to determine the TBB effects on lumbar lordosis and kinematic changes during sitting tasks. Evaluating the TBB usage on LBP subjects with known pathology or recognized conditions may provide insight into how such conditions can be supported and attenuated during normal work settings and as such, this is suggested as an area of future research as is the use of a criterion for comparison.

## Ethical approval

The TBB Study was approved and classified as exempt by the ethics committee of the University of Social Welfare and Rehabilitation Sciences.

## Competing interests

The authors declare that they have no competing interests.

## Funding

This research did not receive any specific grant from funding agencies in the public, commercial, or not-for-profit sectors.

## Authors’ contributions

HRM was involved in the conception and design, analysis of data, drafting of the manuscript, critical revision, and supervision of the study. JG was participated in data acquisition, analysis of data, interpretation of the data and drafting of the manuscript. AMG was involved in the conception and design, analysis and interpretation of data, drafting of the manuscript and critical revision. MA was participated in analysis and interpretation of data, drafting of the manuscript and critical revision for content, administrative support. CPG conceptualized and designed the study, drafted the initial manuscript, and reviewed and revised the manuscript. MP conceptualized and designed the study, supported in analysis of data analyses, and critically reviewed the manuscript for important intellectual content.

## Acknowledgments

The authors like to acknowledge the kind assistance of students who contributed to the research.

**Table 1:** User experience checklist and its descriptive results (n=30)

**Items**		**Strongly agree**	**Agree**	**No comment**	**Disagree**	**Strongly disagree**
1	I thought the belt was easy to use and most people would learn how to use this system very quickly.	33.3%	60%	6.7%	0%	0%
2	I thought the belt was so flexible and adjustable that it suits different people (Adjustability).	26.7%	66.6%	6.7%	0%	0%
3	The belt did not cause motion limitation in the upper limbs during the sitting tasks (ability to move).	36.7%	46.6%	16.7%	0%	0%
4	I thought wearing the belt was easy and not time consuming.	66.6%	30%	3.3%	0%	0%
5	I found the belt safe for contact points on the body.	46.6%	40%	6.7%	0%	0%
6	I felt comfortable while wearing the belt.	36.6%	63.3%	0%	0%	0%
7	I found the belt’s design appropriate for the sitting task.	16.6%	50%	33.3%	0%	0%

**Table 2:** Descriptive data of muscles activity (mV) in the belt and no-belt conditions (mean, SD)

**Time (min)**	**Muscles**											
	**Longissimus**		**Iliocostalis**		**Multifidus**		**Rectus abdominis**		**External oblique**		**Internal oblique**	
	**Belt**	**No belt**	**Belt**	**No belt**	**Belt**	**No belt**	**Belt**	**No belt**	**Belt**	**No belt**	**Belt**	**No belt**
1^th^	5.26 (2.93)	7.26 (3.91)	7.55 (6.89)	6.26 (4.40)	6.22 (4.64)	5.13 (4.12)	1.40 (0.83)	1.49 (0.74)	1.57 (1.09)	2.42 (2.24)	2.28 (2.12)	4.58 (4.10)
5^th^	7.17 (4.16)	7.42 (4.02)	8.62 (7.18)	6.58 (4.75)	7.27 (5.96)	6.43 (4.53)	1.36 (0.78)	1.64 (0.89)	1.47 (0.99)	2.26 (2.00)	2.48 (2.41)	6.76 (2.52)
10^th^	5.64 (3.24)	8.21 (4.89)	7.47 (5.35)	8.19 (6.46)	7.46 (5.92)	7.10 (4.76)	1.38 (0.83)	1.65 (0.98)	1.73 (1.21)	2.03 (1.73)	2.42 (1.98)	4.58 (3.23)
15^th^	4.64 (2.81)	6.44 (4.25)	6.93 (6.47)	6.99 (5.16)	6.39 (6.11)	6.50 (4.52)	1.42 (0.85)	1.59 (1.06)	1.59 (1.08)	1.97 (1.49)	2.93 (2.58)	4.08 (3.50)
20^th^	3.95 (1.91)	6.35 (4.49)	6.38 (5.44)	6.07 (4.35)	6.39 (6.27)	6.10 (4.15)	1.39 (0.81)	1.64 (1.08)	1.71 (1.23)	2.48 (2.05)	2.79 (2.55)	4.33 (3.92)
25^th^	3.75 (1.91)	6.08 (4.50)	5.17 (4.23)	6.57 (5.35)	5.85 (5.43)	6.69 (5.07)	1.44 (0.82)	1.59 (0.95)	1.67 (1.26)	2.38 (2.42)	2.28 (2.44)	4.99 (4.46)
30^th^	3.43 (2.29)	6.70 (4.93)	5.99 (7.07)	5.91 (4.34)	6.58 (7.52)	6.47 (5.42)	1.40 (0.78)	1.71 (0.85)	1.47 (0.94)	2.20 (1.83)	2.58 (2.09)	4.12 (3.75)
Total	4.83 (2.74)	6.92 (4.42)	6.87 (6.08)	6.65 (4.97)	6.59 (5.97)	6.34 (4.65)	1.39 (0.81)	1.61 (0.93)	1.60 (1.11)	2.24 (1.96)	2.53 (2.31)	4.77 (3.64)

**Table 3:** Statistical results of testing the effects of belt and time on para-spinal muscle

**Independent variable**	**Longissimus**		**Iliocostalis**		**Multifidus**		**Rectus abdominis**		**External oblique**		**Internal oblique**	
	***P*** ** value**	**η** ^ 2 ^ ** value**	***P*** ** value**	**η** ^ 2 ^ ** value**	***P*** ** value**	**η** ^ 2 ^ ** value**	***P*** ** value**	**η** ^ 2 ^ ** value**	***P*** ** value**	**η** ^ 2 ^ ** value**	***P*** ** value**	**η** ^ 2 ^ ** value**
Belt	**0.012**	**0.235**	0.313	0.035	0.814	0.002	**0.024**	**0.180**	0.087	0.133	**0.001**	**0.440**
Time	**0.003**	**0.611**	**0.011**	**0.473**	**0.045**	**0.392**	0.629	0.173	0.719	0.186	0.654	0.129
Belt×Time	0.278	0.301	0.216	0.274	0.632	0.154	0.198	0.313	0.650	0.209	0.115	0.466

Activity (RMS) determine by using repeated measures ANOVA. Note: Bold values indicate significant effects, *P* < 0.05.
